# Reflections on Some of the Exceptional Features of HTLV-1 and HTLV-1 Research: A Perspective

**DOI:** 10.3389/fimmu.2022.859654

**Published:** 2022-04-01

**Authors:** Robert C. Gallo, Yutaka Tagaya

**Affiliations:** ^1^Institute of Human Virology, University of Maryland School of Medicine, Baltimore, MD, United States; ^2^Cell Biology Lab, Division of Virology, Pathogenesis and Cancer, Institute of Human Virology, University of Maryland School of Medicine, Baltimore, MD, United States

**Keywords:** HTLV-1, human retrovirus, human oncovirus, leukemogenesis, development of a concept

## Abstract

The report is not a review or a summary. In a manner, it is a perspective but an unusual one. It looks back to the years my colleagues and I (RG) began preparing for human retroviruses (beginning in 1970), how they evolved, and attempts to bring to light or simply to emphasize many exceptional characteristics of a retrovirus known as HTLV-1 and some fortuitous coincidences, with emphasis on the needs of the field. These events cover over one half a century. We have had many reviews on HTLV-1 disease, epidemiology, and basic aspects of its replication, genome, gene functions, structure, and pathogenesis, though continued updates are needed. However, some of its truly exceptional features have not been highlighted, or at least not in a comprehensive manner. This article attempts to do so.

## Lack of Adequate Attention

The first unusual feature mentioned is not exactly science-based, but it has been a recurrent one. The first submitted encompassing major manuscript describing this cancer virus (to be known as HTLV-1 later), the first known human leukemia virus, and the first human retrovirus was immediately rejected by the *Journal of Virology* with hostile comments from the editor that more or less concluded that everyone knows there are no human retroviruses and such reports, even such work, must stop.

Thankfully, there was the *U.S. Proceedings of the National Academies of Science*, which accepted the first few papers (1, 2), as did *Nature* (3). Not the least of these was the timing. The 1950s–1960s were years of acceptance of the possibility and even probability of finding human retroviruses. However, by the 1970s, there was a decline in interest in serious infectious diseases even to the point of the minimization of departments of microbiology and promotion of investment of funding to non-infectious “degenerative diseases”. Some of the reasons for a particular bias against human retroviruses are summarized in **Table 1**. Not included on the list was the reaction against claims made for detecting human retroviruses in many human cancers purely by a biochemical approach. Many publications appeared from a leading virology lab in NY on this subject (4), but were not substantiated. However, my colleagues and I (RG) soon showed that these were false positives, and their detection of a “retrovirus reverse transcriptase” was, in fact, DNA polymerase gamma, the mitochondrial DNA polymerase (5, 6).

**Table 1:** Some considerations why resistance and bias against even the possibility of human retroviruses were prevalent in the 1970s.

A strong search for them funded by the U.S. National Cancer Institute (NCI) virus cancer program was unsuccessful in the 1950s to late1970s. Evidence that the well-studied animal retroviruses replicated to high titers when causing disease. The assumption then was that human retroviruses would be easy to discover if they existed. No sensitive tests would be needed. Human sera were lytic against complement-destroyed retroviruses (small animal retroviruses were used in this study), which led to the assumption that humans were, therefore, protected against retroviruses, in fact, not against human and some primate retroviruses. Several false starts. In particular, many claims were made from a biochemical approach to detecting human retroviruses in a number of different human cancers, but their assays were not specific and misinterpreted. It was thought that the concept of infectious causes of cancer was a primitive notion that implied that one could “catch” cancer. The assumption is based on acute viruses, but not considered were “slow viruses”, among which are retroviruses.

In the 1970s, there were also very strong biases against any role for any infectious agents in any human cancer. Of course, these biases came tumbling down by the 1980s, as human retroviruses were discovered, shown to cause human diseases, and overall, along with other human tumor viruses, a role for viruses in human cancer was estimated to be about 20%. Soon after we discovered the second human retrovirus, HTLV-2 (7, 8), the third human retrovirus (9–13) was now on “top” of us and causing, oddly enough, our first pandemic in some decades, AIDS (14, 15), a disease, if left untreated, would have killed almost all within a few years.

Though HIV has long held a prominent position (by necessity), attention to HTLV-1 quickly faded. This includes both knowledge about it and research support for it. This is not easy to understand. Though HTLV-1 is not widespread in the United States, most of Europe, China, and India, which form the largest centers for publicity and most of the world science support, it is very prominent in select places such as Japan; in Native Australians; in parts of Indonesia, Romania, and Iran; in several countries in South America but especially Peru and Brazil; in parts of equatorial Africa and migrants originating from Africa to Europe; in the United States; and especially in the islands of the Caribbean (16). The oddity of this distribution is itself fascinating but may be explained by the poor transmissibility of HTLV-1 and its tendency to remain in families and certain populations, making the virus a useful aid in historical demography (17, 18).

## Discoveries of Human Retroviruses

One of us (RG) decided on two simultaneous efforts in a search for human retroviruses. One was to develop a sure surrogate marker for a retrovirus far more sensitive than electron microscopy because the titer of any putative retrovirus, if present, must be very low because of difficulty in finding one, a situation very different from the well-studied and well-known animal retroviruses that replicated to high levels. At the same time, we wanted this surrogate assay to allow frequent sampling of cultures because the virus may be expressed only in a “pulse”. Both were true, and our selection proved worthy. Indeed, very recent findings suggest that the plus-strand of the HTLV-1 provirus including tax remains transcriptionally silent, and only intermittently as bursts by cellular stress such as hypoxia and glycolysis (19–23). In 1970, reverse transcriptase (RT), the soon to be well-known DNA polymerase of retroviruses, was independently discovered by Howard Temin and David Baltimore. It had provided important support for Temin’s provirus hypothesis, a key tool in some gene cloning efforts, and for me, the needed surrogate for a retrovirus.

After years of studies, we could make retrovirus assay sensitive (×1,000 greater than electron microscopy) [the detail of the assay and its development was reviewed in ref (24)], readily applicable to the frequent sampling of cultures, and specific, i.e., distinguishable from the multiple human cellular DNA polymerases (5, 6, 24, 25). Among advances from others contributing to this were developments from a biotech company, Collaborative Research Inc., and the collaboration of their CEO, Ori Firdieman, with Baltimore in developing synthetic homopolymeric/oligomeric template/primers. We tested various forms of them over the years in the 1970s, and some were critical.

The second approach was attempted to grow primary human blood cells in sufficient amounts and over sufficient time to attempt proper culture. This would require growth factors, and a few were only recently discovered from conditional media. One key result we made was the discovery of a T-cell growth factor, later termed interleukin-2 (IL-2), which became the first studied cytokine (26–28). During the 1970s, the first infectious leukemia causing retroviruses of primates was discovered by Takeshi Kawakami in a Gibbon Ape (29), and in 1978, we found a new variant associated with T-cell leukemia among Gibbons in the wild (30). This Gibbon Ape T-cell leukemia virus coupled with IL-2 pushed us toward greater concentration on human T-cell leukemias, and with the good fortune of a thorough and dedicated post-doctoral working in my group, the key results were obtained (1–3, 7, 31–34).

Soon we found the second human retrovirus, HTLV-2, in a leukemic cell from a patient with a T-cell variant of having hairy cell leukemia (7). This virus is much less pathogenic than HTLV-1, differs mainly in some parts of the 3′ region of the genome, and likely had a different primate-to-man origin (8).

## Exceptional Oncogenicity

It is estimated that HTLV-1 causes cancer in about 5% to 6% of infected people (16, 35, 36), but if the infection is acquired during infancy from, for example, mother’s milk, it may be as high as 20% (36). Either number is an exceptionally high rate of cancer causation from a single exposure to a carcinogen. This means the risk from HTLV-1 to cancer is one of the most dangerous pathogens known (35), but as we will mention later, HTLV-1 also causes other diseases. Another extraordinary feature of HTLV-1 is oncogenicity as proved by the amount of evidence available for its causative role summarized in **Table 2** and the lack of any identifiable co-factors like HIV and AIDS—the virus and its human target seem sufficient. However, that does not mean there are no factors that promote the likelihood of cancer development. There may be many other factors limiting its oncogenicity, but to date, and unlike many other carcinogens, there is no known strong co-factor necessary for causation. This point is rather forcefully drawn home by *in vitro* transformation and *in vivo* transfection studies that lead to transformed cells that look and act like human adult T-cell leukemia/lymphoma (ATL) cells. Alone among known tumor viruses, HTLV-1 infection apparently does it all. Sometimes, the issue has been raised as to why so many do not get leukemia when infected. My hypothesis is that the HTLV-1 provirus drives the proliferation of infected cells for its survival since HTLV-1 is so poorly infectious and transmission comes mostly from infected cells. It is also plausible that the establishment of the *in vivo* leukemia may require the accumulation of somatic mutations in infected T cells, which is driven by the clastogenic nature of HTLV-1 with time. Recent whole-genome analyses of the ATL cells from many patients indeed demonstrate convergent patterns of genetic mutations (37–39). It is also suggested that aggressive (acute/lymphomatous subtypes) ATL is associated with an increased burden of genetic and epigenetic alterations (39), demonstrating the cumulative nature of genetic alterations in advancing the phenotype of ATL. Another recent finding that may corroborate this point is that ATL seems to be the leading cause of death even among human T lymphotropic virus type I-associated myelopathy/tropical spastic paraparesis (HAM/TSP) patients (40), suggesting that the process of cellular transformation by HTLV-1 indeed takes a long time but keeps accumulating while HTLV-1-infected cells manifest non-leukemic disorders.

**Table 2:** Exceptional linkage of HTLV-1 to adult T-cell leukemia/lymphoma (ATL).

It is the same kind of virus causing leukemia in many different animals. It is epidemiologically linked to one particular leukemia/lymphoma, ATL. Infection of normal T cells *in vitro* leads to their immortalized growth and leukemia transformation. The HTLV-1 provirus is clonally integrated into ATL cells obtained from patients, demonstrating that the leukemia cells were infected before the leukemic transformation. Transfection of the HTLV-1 provirus or the transgene expression of either of two HTLV-1 specific genes (*hbz*, or *tax*, under T-cell specific promoters) leads to a T-cell leukemia/lymphoma development in mice. Molecular studies *in vitro* reveal mechanism of neoplastic transformation.

Perhaps it is just a matter of time. The median value for the time required for HTLV-1-infected cells to accumulate sufficient mutations to become leukemic may be larger than the average life of humans. Given a far greater life span, we suspect that transformation might occur in most infected persons. So it is interesting to raise the question as to whether the early formation of the ATL cells is multifactorial as human cancer formation is in general or if HTLV-1 does it all. Also, is there any co-factor that might accelerate/diminish the speed of genetic alterations in HTLV-1-infected cells? **Table 2** lists the exceptional evidence showing that HTLV-1 causes leukemia.

## A Challenge to the Prevailing Dogma: One Gene Sequence→One Protein. One Protein→One Function. One Agent (Like a Virus)→One Disease

These were simple statements many of us heard when were still students, but, of course, as we have learned, none are true. I cannot think of any situation where these are all illustrated more clearly than HTLV-1 infection and disease. HTLV-1 is the first virus where multiple splicing of its primary RNA transcript was demonstrated (41), revealing that one genetic sequence could and indeed regularly contribute to the encoding of several proteins.

So too, some of its proteins unexpectedly reveal functions beyond the main and obvious one. For example, we know tax protein not only has trans-acting transcriptional activity but may be released from cells and have effects on other (by-stander) cells (42, 43), or the HBZ factor, which contributes to neoplastic transformation, impacting cell growth prior to transformation as well as controlling inflammation as protein and RNA (44–50).

Above, it was already noted that HTLV-1 causes more than ATL. It also induces spastic paralytic disease (HAM/TSP, HTLV-1 associated myelopathy/Tropical spastic paraparesis) immune mechanisms (51–54). Additionally, a slight variant from more common forms of HTLV-1 and known as HTLV-1c causes severe bronchiectasis, impacting the lives of native Australians (55–59). Several less crippling (yet are public health burden) disorders (including dermatitis and uveitis) are also associated with HTLV-1 (60–62). Infectious dermatitis is drawing clinical attention due to its manifestation among children and because it could develop into ATL or HAM/TSP later (63, 64). Overall, about 10% of HTLV-1 infections lead to multiple diseases.

## HTLV Research Leads to HIV Discovery: An Unusual Coincidence of Their Discoveries Coming Just Before AIDS Was First Recognized

As noted above, in the 1970s, there was broad and strong resistance even to the possible existence of human retroviruses. The discoveries of the HTLVs (HTLV-1 reported in 1980 and HTLV-2 in 1982) were just being accepted when AIDS was first recognized in 1981, opening up the idea that AIDS might have a similar origin. However, by the time a patient was recognized as having AIDS, usually, the patient had many microbial infections. The question was which one was the cause? It turned out to be quite challenging; a hidden retrovirus was suggested and shown to be the cause. We can say with confidence that without the HTLV experience, this would have been thought of as a wild and unacceptable notion. Further, the key technology utilized for the discovery of HIV came out of HTLV research, namely, the growth of primary human blood T cells with IL-2 and a very sensitive and specific assay for a retrovirus (proper assays of RT), which were key to the findings on HIV.

## HTLVs as Model Systems

We think of studies of HTLVs as significant not only for the cancer it causes but also for lessons provided from studies on the mechanisms that can cause spastic paralysis or immune disorders. There are many ways of exploiting HTLV infections to these ends. *In vitro* transformation of umbilical cord T cells (65) through co-culture with patient-derived ATL cells and the establishment of ATL cell lines from ATL patients (66, 67) were the first step that enabled the extensive research on how HTLV-1 transforms host cells. The cells display full ATL-like cancer characteristics (cellular and molecular features as well as their capacity to grow in animal models to form ATL-like leukemia/lymphoma), and the mechanisms underlying the cellular transformation by HTLV-1 have been widely studied using these ATL cell lines, to give rise to quite a few publications. This, in turn, has been made into *in vivo* models by expressing the HTLV-1 *tax* (68, 69) or *hbz* (45) genes as a transgene in mice. Both tumors and central nervous system (CNS) animal models are available (70). A prime example of a clinical advance to unrelated diseases came out of a drug development program planned as a limitation on CNS attack by immune cells and by drug developments against ATL. One of these drugs that we are developing (BNZ-1) is now undergoing trials for a similar CD4 T-cell malignancy (cutaneous T-cell lymphoma), which is not directly related to HTLV-1 (71, 72). As to the bronchiectasis/pulmonary disease frequently observed among native Australians (55, 56, 58, 59), this may have something to do with the unique origin and divergent DNA sequence of the HTLV-1c (57) compared with globally dominant HTLV-1 strains. Of interest is that the same region adjacent to the 3’ end of the viral genome is what chiefly distinguishes HTLV-1 from the less pathogenic HTLV-2 (8), suggesting that this region of HTLVs may control the pathogenesis of individual HTLV strains/subtypes. It will, of course, be most interesting to define how differences in sequences among HTLVs affect their pathogenesis, if they do.

There is a need for serious funding programs aimed at obtaining effective therapy and preventive vaccine for HTLV-1. This must be done in a climate of much greater attention and appreciation of the field along with greater funding.

## Where HTLV Research Might Focus

First, more attention to the field is needed. This can only come from strong promotion, as has been carried out by Fabiola Martin for HTLV-1 research in Australia and in discussions with WHO (73, 74), but all in the field can do more. Those in areas of the world that are hardest hit by HTLV-1 are primed to be the most important activists. At present, this is in South America, Japan, and the native population of Australia. Eduardo Gotuzzo of Peru has been one such leader from South America, as have Toshi Watanabe, Yoshi Yamano, and Masao Matsuoka for Japan. Iran and Romania are enigmas. Hopefully, we will soon learn more about their HTLV-1 cases and research from “neighbors” like Ali Bazarbachi of Lebanon and with close ties with outstanding groups in Europe, notably the one led by Charles Bangham in London and several Italian groups including the one led by Roberto Accolla and colleagues.

Clearly understudied and underfunded are efforts directed to developing effective therapy and a preventive vaccine. We are hopeful that this will soon change.

## Author Contributions

RGC and YT wrote the paper together. All authors listed have made a substantial, direct, and intellectual contributions to the work and approved it for publication.

## Funding

This work was supported by the departmental funding of the Institute of Human Virology at the University of Maryland School of Medicine, Baltimore.

## Conflict of Interest

YT holds financial interests with BIONIZ Therapeutics LLC, now Equillium Inc., (San Diego, CA) which holds the patent and intellectual property for the BNZ-1 peptide mentioned in this article.

The remaining author declare that the research was conducted in the absence of any commercial or financial relationships that could be construed as a potential conflict of interest.

## Publisher’s Note

All claims expressed in this article are solely those of the authors and do not necessarily represent those of their affiliated organizations, or those of the publisher, the editors and the reviewers. Any product that may be evaluated in this article, or claim that may be made by its manufacturer, is not guaranteed or endorsed by the publisher.
